# A Modular Cloning Toolbox for the Generation of Chloroplast Transformation Vectors

**DOI:** 10.1371/journal.pone.0110222

**Published:** 2014-10-10

**Authors:** Yavar Vafaee, Agata Staniek, Maria Mancheno-Solano, Heribert Warzecha

**Affiliations:** Plant Biotechnology and Metabolic Engineering, Technische Universität Darmstadt, Darmstadt, Germany; Imperial College London, United Kingdom

## Abstract

Plastid transformation is a powerful tool for basic research, but also for the generation of stable genetically engineered plants producing recombinant proteins at high levels or for metabolic engineering purposes. However, due to the genetic makeup of plastids and the distinct features of the transformation process, vector design, and the use of specific genetic elements, a large set of basic transformation vectors is required, making cloning a tedious and time-consuming effort. Here, we describe the adoption of standardized modular cloning (GoldenBraid) to the design and assembly of the full spectrum of plastid transformation vectors. The modular design of genetic elements allows straightforward and time-efficient build-up of transcriptional units as well as construction of vectors targeting any homologous recombination site of choice. In a three-level assembly process, we established a vector fostering gene expression and formation of griffithsin, a potential viral entry inhibitor and HIV prophylactic, in the plastids of tobacco. Successful transformation as well as transcript and protein production could be shown. In concert with the aforesaid endeavor, a set of modules facilitating plastid transformation was generated, thus augmenting the GoldenBraid toolbox. In short, the work presented in this study enables efficient application of synthetic biology methods to plastid transformation in plants.

## Introduction

Although the majority of genetically engineered plants today are generated by integrating transgenes into the nuclear genome, engineering of the plastid genome has become a promising technology, both for basic science and applied plant biotechnology [1]. Their potential for successful genetic manipulation stems from the fact that plastids, as relicts of endosymbiotic cyanobacteria, still feature many characteristics of prokaryotes. First, genome modulation can be achieved easily due to the still present and efficiently functioning homologous recombination system. By selecting stretches of plastid DNA to flank any given sequence, transgenes can be integrated into the plastid genome at virtually any location, enabling both mutagenesis of endogenous sequences and incorporation of additional genes with very high efficiency. Second, cells harbor a multitude of plastids, especially chloroplasts; these, in turn, carry multiple genome reprints. This high (trans-) gene copy number per cell, coupled with the utilization of strong promoters, fosters significantly elevated expression rates resulting in unprecedented protein accumulation levels (e.g., 80% TSP for bacteriolysins [2]). Last but not least, in many plant species, plastids are exclusively inherited maternally. This could be considered a built-in genetic containment feature, as the spread of transgenes by pollen is, consequently, largely excluded.

The enumerated traits constitute clear benefits for molecular farming, wherein the expression of one or a few transgenes at maximum levels and the safety of open field applications are major goals. But plastid engineering boasts still more potential that has come into focus only recently: the prospect of multigene stacking and coordinated gene expression for metabolic pathway engineering and the unmitigated access to reducing power from photosynthetic processes, conceivably enabling light-driven generation of metabolites. Polycistronic organization of plastidic operons, affording synchronized expression of multiple genes driven by a single promoter, is a well-established phenomenon [3]. Yet only recently, the group of Ralph Bock has taken advantage of this feature and designed a multigene operon for the concerted expression of three biosynthetic genes leading to the formation of tocochromanols in tomato [4]. The resulting study provides an excellent example of the advantages of plastid engineering for the build-up of metabolic pathways, affording enhanced levels of natural product retrieval. In another very recent report, Lassen *et al*. showed that cytochrome P450 enzymes, requiring electrons (usually delivered from NADPH by an accompanying reductase) for their inherent transformation reactions, can be coupled to the photosynthetic electron translocation machinery within the chloroplasts [5]. Since P450s are important catalysts in numerous biosynthetic routes leading to the formation of valuable natural compounds [6], metabolic engineering within the chloroplasts promises to foster the build-up of efficient pathways fueling light-driven biosynthesis of alternative metabolites.

Despite the numerous advantages of plastid transformation, some persisting bottlenecks and drawbacks still hamper many potential applications. Most discouragingly, not all plant species are amenable to the technique, with the monocotyledons, including agronomically important grasses like rice or maize, proving especially problematic. Although many successful transformation protocols of various plant species have been published (e.g., tomato [7], lettuce [8], or sugar beet [9], to name only a few), only tobacco and, to some extent, tomato and lettuce, as well as the unicellular algae *Chlamydomonas reinhardtii* can be routinely transformed with reasonable effort. Furthermore, identification of a large set of promoters, terminators, and regulatory elements driving the expression of plastid transgenes notwithstanding, the most suitable combination of the aforementioned sequences for any given transgene is hard to predict, as is the stability of the resulting recombinant protein. For example, while the 5′- and 3′-transcript untranslated regions (UTRs) substantively bear upon RNA stability [10], the 5′-segments of coding sequences (CDSs) significantly influence translation efficiency [11], [12]. Hence, rational and targeted manipulation of the aforesaid genetic elements can substantially boost expression levels. Moreover, it has been shown that N-terminal fusions of short peptides as well as signal sequences directly affect the stability of recombinant proteins [13]. In light of all the enumerated findings pertaining to the influence of diverse sequence elements on gene expression and protein stability, construction of large sets of transformation vectors becomes a prerequisite for effective modification of the plastid genome. Taken together with the inherent requirement to replace and shuffle flanking sequences necessary for homologous recombination and integration of the transgene cassettes into the genome of a given plant at a specific position, the cited considerations point to extensive and oftentimes cumbersome cloning procedures as the critical hurdle to dynamic development and far-reaching application of plastid genome engineering. Consequently, while an extensive array of expression vectors have been developed and made available to the research community in recent years [14]–[16], the engineering of novel genetic elements and target plant species still requires tedious redesign and recloning in almost all cases. On the one hand, with DNA synthesis becoming less and less expensive, the challenge can now be addressed through total synthesis of optimized vectors, eliminating repetitive sequences or unfavorable restriction sites by design. On the other, the synthetic approach provides merely case-by-case solutions to the individual experimental objectives and is at odds with the central premise of rational bio-engineering.

Standardization of reusable biological components as a means to efficiently design and engineer biological systems is a paradigm of synthetic biology, as recently reiterated by one of its co-founders [17]. While in many ways the young discipline has been staggeringly successful, with the creation of the minimal cell marking a stepping stone in its ground-breaking advent [18], the very concept of standardization – the driving force of Industrial Revolution and primer of the Information Age shaping modern society [19] – still lacks universal validation in the field of biological engineering.

The first attempt at the development of a standardized strategy for combinatorial manipulation of DNA fragments was reported nearly two decades ago [20]. Although versatile and elegant, NOMAD (nucleic acid ordered assembly with directionality) met with but limited acceptance within the scientific community, while the *ad hoc* experimental design of DNA assembly efforts persisted. In contrast, the BioBrick standard [21], launched in concert with the International Genetically Engineered Machines competition (iGEM), garnered considerable traction and spurred exuberant development of “standard biological parts” and their applications [22]. While certainly tantalizing, the simplicity of the iterative BioBrick approach turned out to be one of its limitations, as the original design, burdened with the obligatory by-product of residual scarring between individual parts, does not translate into the higher orders of abstraction – beyond genes, into pathways and coordinated circuits. In response to the system constraints, an array of alternative DNA assembly methods have been developed and critically reviewed, addressing their prospective application in both microbial and plant engineering [23], [24].

Among others, the expanding toolbox of synthetic biology offers a powerful technology dubbed Golden Gate [25]. Drawing on the distinct properties of type IIs restriction enzymes, the strategy affords multipartite and seamless (or scar-benign) assembly of genetic elements in a “one pot, one step reaction” [26]. In turn, the founding principle of Golden Gate precision cloning proved the corner stone of the concurrent development of two standardized modular cloning systems, MoClo [27] and GoldenBraid, GB [28]. Further coordinated efforts rendered the two compatible and ultimately resulted in the introduction of the common assembly standard for plant synthetic biology, GoldenBraid 2.0 [29], offering its users a starter kit of ready-made genetic modules as well as relevant software tools (https://gbcloning.org/).

The GB 2.0 destination plasmid kit encompasses two complimentary sets of binary vectors based on the pGreenII and pCAMBIA vector backbones, respectively. Thus, the original system solely addresses the *Agrobacterium tumefaciens*-mediated transfer of foreign DNA into the plant cell nucleus. To further establish GoldenBraid as the modular cloning system overarching the full spectrum of plant genetic engineering, we demonstrate its reappropriation for plastid transformation. The proposed comprehensive application of the GoldenBraid grammar will afford straightforward and seamless assembly of coordinated fusions (e.g., promoter-UTR) and multigene operons compatible with the genetic machinery of chloroplasts. It will further allow utilization and effortless shuffling of relevant flanking regions characteristic of not only different parts of a specific plastid genome, but indeed, those of diverse representatives of the plant kingdom, thus enabling easy adjustment to alternative species. Furthermore, bolstering the GB toolbox will foster free exchange of the standardized parts between the nuclear- and plastid-specific transformation vectors. The across-the-board compatibility of the GoldenBraid system thus ensured boasts the potential for prospective establishment of an ever-expanding repository of reusable genetic components and bringing together multiple users within the plant scientific community.

## Materials and Methods

### Cloning of GB parts (domestication)

All DNA fragments were amplified by PCR using corresponding templates (either plasmid DNA or genomic DNA from tobacco or lettuce) and high fidelity DNA polymerase (Thermo Scientific, St Leon-Roth, Germany) based on the protocol provided by the manufacturer. The DNA sequence encoding griffthsin was ordered as a synthetic gene from Thermo Scientific (St Leon-Roth, Germany) and the primers were obtained from Eurofins MWG GmbH (Ebersberg, Germany). All primers were designed so that they contained the appropriate *Bsm*BI restriction sites and overhangs to be subsequently cloned into the universal domesticator vector (pUPD) [28]. All overhangs released upon *Bsa*I-cleavage of pUPD constructs were designed to give the parts the appropriate identity (e.g., promoter, CDS, etc.). Only pUPD containing left and right targeting regions (LTR and RTR, respectively) were flanked by GGAG at the 5′-end and CGCT at the 3′-end to enable their cloning as single fragments into any α-level pDGB vector.

For templates containing internal type IIs recognition sites (*Bsa*I, *Bsm*BI, and *Bbs*I), additional primers were designed, allowing amplification of the given part in two or more patches (according to [27]). The patch-flanking *Bsm*BI-cleavable overhangs facilitated in-frame fusion of patches, resulting in parts with point mutations, removing the unfavorable recognition sequences. Ω vectors conferring chloramphenicol resistance were assembled directly from PCR products of backbone parts with compatible overhangs cleaved by *Bbs*I in digestion/ligation GB reactions (see below). The chloramphenicol resistance gene (*cat*), including the appropriate promoter and terminator, was amplified in two patches from the vector pSB1C3 (Biobrick registry part, http://parts.igem.org), while the ori and flanking regions were PCR-synthesized from pICH41306 [27]. GB cassettes with the *lacZ* gene were recloned from the appropriate pDGB1_Ω vectors [28]. The newly assembled vectors were provisionally termed pDGB3_Ω.

PCR products used in the GoldenBraid reactions were purified by QIAquick PCR Purification Kit (Qiagen, Hilden, Germany). Standard GB reactions were set up in 10 µl mixtures containing 75 ng of the target vector, 75 ng of the PCR products (GB parts or patches) or intermediate vectors carrying corresponding fragments, T4 DNA ligase buffer (Promega, Mannheim, Germany), 3 U of the required restriction enzyme (*Bsa*I or *Bsm*BI), and 1 U of T4 DNA ligase (Promega, Mannheim, Germany). The assembly reactions were performed as 25 cycle digestion/ligation reactions (2 min at 37°C, 5 min at 16°C). One µl of each GB reaction mixture was transformed into chemically competent *E. coli* Top10 cells. Positive clones were selected on LB plates containing ampicillin (for the domestication vectors), kanamycin (for α-level destination vectors), and chloramphenicol (for pDGB3_Ω destination vectors). Blue/white selections were performed on plates supplemented with 50 µl X-Gal (2% (w/v) in DMSO) prior to plating. Plasmid DNA preparations were made using the E.Z.N.A. Plasmid Mini Kit I (Omega Bio-Tek, VWR, Darmstadt, Germany). Correct assemblies were confirmed by plasmid restriction analysis using *Eco*RI (pDGB1_α1 and pDGB3_Ω1R), *Bam*HI (pDGB1_α1R and pDGB3_Ω1), *Hind*III (pDGB1_α2 and pDGB3_Ω2R), and *Eco*RV (pDGB1_α2R and pDGB3_Ω2). *Bsa*I was provided by New England Biolabs (Ipswitch, MA, USA). All remaining restriction enzymes were purchased from Fermentas (Thermo Scientific, St Leon-Roth, Germany).

### Cloning in α- and Ω-level destination vectors

After assembly of all parts in pUPD vectors, the relevant transcriptional units (TU) were generated in α-level destination vectors (pDGB1). The pDGB1 vectors are derived from pGreenII binary vectors [30], reconstructed and adapted for the GB cloning system by Sarrion-Perdigones *et al.* [28], [29].

The 2000 bp stretch of the *Nicotiana tabacum rbcL* gene in pUPD, serving as the left targeting region (LTR) of the expression cassette, was cloned into the pDGB1_α1 vector. In parallel, the aminoglycoside 3′-adenyltransferase (*aadA*) coding sequence was assembled with the *N. tabacum rrn* promoter (*Nt*P*rrn*) and the terminator of the *N. tabacum psbA* gene (*Nt*T*psbA*) into the pDGB1_α2 vector, yielding the TU*aadA*. The *Bsa*I-GB reactions were performed as 25 cycle digestion/ligation reactions (2 min at 37°C, 5 min at 16°C). After transformation into *E. coli*, positive clones were selected on plates containing kanamycin and extracted plasmids were used as templates for subsequent cloning steps. TU*aadA* and LTR were then combined in the pDGB3_Ω1 vector in a *Bsm*BI-GB reaction, yielding pDGB3_Ω1:LTR-TU*aadA*. In parallel, the RTR (2000 bp of the *N. tabacum accD* gene) was cloned into the pDGB1_α2 vector and the coding sequence of griffithsin was combined with the *N. tabacum psbA* promoter (*Nt*P*psbA*) and the *N. tabacum psbA* terminator (*Nt*T*psbA*) into pDGB1_α1R, yielding TU*GRFT*. RTR and TU*GRFT* were then combined in pDGB3_Ω2, yielding pDGB3_Ω2:RTR-TU*GRFT*. Both pDGB3 (Ω1 and Ω2) constructs were then combined in pDGB1_α2, yielding the final transformation vector.

Verification of the final tobacco chloroplast transformation vector was performed via PCR amplification of its various components (including promoters, CDSs, terminators, and flanking sequences) as well as restriction enzyme digestion.

### Chloroplast bombardment and molecular analysis

*N. tabacum* (cv. petit havana) leaf explants were bombarded with a BioRad PDS1000 (He) gene gun (BioRad, Hercules, CA, USA), as described previously [31] and placed upside down on RMOP medium containing 500 mg×L^−1^ spectinomycin under a 16 h light and 8 h dark photoperiod at 25°C in a cultivation room. To confirm integration of the transgene in the tobacco plastome, DNA preparations of developing green plantlets were tested by PCR with the corresponding primers. To obtain homoplasmy, positive transgenic shoots were subjected to two to three additional rounds of regeneration. Homoplasmic shoots were transferred to the rooting medium (MS containing 500 mg×L^−1^ spectinomycin) under standard cultivation room conditions. Acclimatization was performed by placing the transplastomic plants under a transparent plastic hood for three days in the greenhouse with 16 h of illumination per day.

For Southern blot analysis, total DNA was extracted from transplastomic tobacco lines based on a previously described protocol [32]. The DNA (2 µg) was digested with *Eco*RI for 16 h; the resulting fragments were separated at 25 V on a 1% agarose gel, and transferred to nylon membranes (Roth, Karlsruhe, Germany). A 1 kb DIG-labeled probe was amplified using the PCR-DIG Probe Synthesis Kit (Roche Molecular Biochemicals, Mannheim, Germany). After hybridization at 42°C for 16 h, the membranes were washed with 2×SSC buffer for 15 min and 0.5×SSC buffer for 30 min at room temperature. Probe-target hybrids were detected with alkaline phosphatase conjugated antibody through a color reaction with NBT/BCIP as a substrate (Roche Diagnostics, Mannheim, Germany). For northern blot, RNA extraction was performed as previously described [33]. For each sample, 2 µg of RNA were electrophoretically separated, the gels blotted and hybridized with the DIG-labeled *griffithsin*-specific probe (amplified and detected as before).

Protein detection was carried out by western blotting. Leaf proteins were extracted in 3 volumes (v/w) of 2×SDS sample buffer (100 mM Tris-HCl pH 6.8, 4% SDS, 0.2% bromophenol blue, 20% glycerol), separated by SDS-PAGE (separating gel buffer, 1.5 M Tris-HCl pH 8.8; stacking gel buffer, 0.5 M Tris-HCl pH 6.8; running buffer, 0.025 M Tris base, 0.2 M glycin, 0.1% SDS (w/v); 12.5% acrylamide; PerfectBlue Dual Gel System, PeqLab, Erlangen, Germany), and transferred to a PVDF membrane (Roth, Karlsruhe, Germany) by wet blotting (PerfectBlue Tank Electro Blotter Web S, PeqLab, Erlangen, Germany) according to the manufacturer’s instructions. For the detection of the His-tagged griffithsin, the membrane was incubated with the His-probe mouse monoclonal IgG_1_ (1∶10000 in PBST) and the goat anti-mouse IgG-HRP (1∶20000 in PBST) as, respectively, the primary and secondary antibodies (Santa Cruz Biotechnology, Heidelberg, Germany). The blot was developed through application of the CheLuminate-HRP Pico Detect Reagent (AppliChem, Gatersleben, Germany) and visualized by exposure to an X-ray film (Fujifilm Corporation, Tokyo, Japan).

## Results and Discussion

The GoldenBraid 2.0 assembly system relies on a series of alpha (α) and omega (Ω) vectors facilitating iterative stacking of transcriptional units. Although the GB vectors are binary T-DNA vectors based on either pGreen (pDGB1) or pCAMBIA (pDGB2) backbones designed for nuclear transformation, they are, in principle, suitable for the assembly of plastid transformation vectors as well. However, the choice of the antibiotic resistance marker in the Ω vectors – the *aadA* gene conferring spectinomycin resistance – counteracts the assembly of standard plastid transformation vectors. Since many plastid expression cassettes contain the *aadA* gene under the control of the *rrn* promoter conjointly functional in *E. coli*, this feature will confer resistance to spectinomycin to all vectors containing the *aadA* transcriptional unit, making it impossible to select assembled Ω vectors from the preceding α vectors. Therefore, we decided to generate a new series of Ω vectors (provisionally dubbed pDGB3_Ω) containing an alternative resistance marker, the chloramphenicol acetyl transferase gene (*cat*). As the new series of Ω vectors for plastid module assembly do not need to contain T-DNA elements, we designed minimal vectors encompassing the *cat* gene, the pMB1 origin of replication, as well as the *lacZ* gene flanked by the appropriate GB 2.0 assembly sites. As the α-level vectors in their original form are amenable for plastid modular cloning, they were not modified.

Next, we wished to include the flanking regions for double homologous recombination into the modular build-up of the expression cassettes to enable maximal flexibility of integration sites within a given plastid genome as well as plant species to be targeted. For this feasibility study, we chose the intergenic region between the *rbcL* and the *accD* genes, proven efficient in previous studies [34], of tobacco and lettuce. Due to the fact that all GB 2.0 (and MoClo) compatible elements have to be devoid of internal type IIs restriction sites (specifically, *Bsa*I, *Bsm*BI, and *Bbs*I), targeting regions needed to be domesticated and the recognition sites removed. Protein encoding sequences, like *rbcL* and *accD*, can be easily mutated (silent mutations) without altering gene functionality. There are other frequently used intergenic regions that might have advantages over this particular site, like the *trnfM* and *trnG* intergenic region [7]. However, mutagenesis of RNA encoding or regulatory sequences might have detrimental effects on the function of these endogenous genes, which needs to be evaluated case-by-case. The selected flanking regions were designed to be incorporated into the universal domesticator plasmid (pUPD) with GB overhangs 1 and 2 [29], enabling subsequent integration into any α vector, depending on the cloning and assembly strategies.

**Figure 1:**
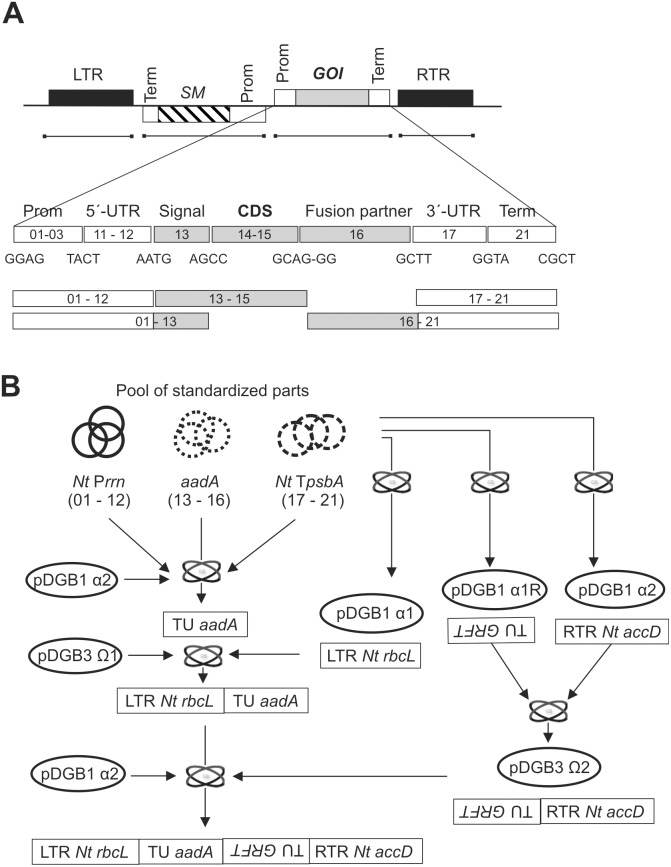
Schematic representation of the modular build-up and the overall GB-based cloning strategy of expression vectors. **A)** Generic structure of a plastid transformation vector. Magnified details show the modular build-up of a transcriptional unit and how it can be assembled from a set of standardized parts. Numbers within the boxes represent part identity and compatibility. Prom, promoter; Term, terminator; LTR, left targeting region; RTR, right targeting region; UTR, untranslated region; SM, selection marker; GOI, gene of interest; CDS, coding sequence. **B)** Schematic representation of the cloning strategy yielding the expression vector used in this study. In the pool of standardized parts, circles represent pUPD vectors harboring genetic elements (parts). Elliptical structures represent the different α- and Ω-vectors. Two intertwined ellipses on an arrow represent a one-pot restriction/ligation reaction (GB reaction) combining all the relevant parts. Boxes represent the parts and their assembly.

For testing of the GB approach in plastid transformation, we designed and cloned a set of genetic elements to be used in transcriptional unit (TU) assembly. Promoters were designed to encompass 5′-UTRs and terminators, to contain 3′-UTRs and stabilizing elements. Considering the terminology proposed in [29], our strategy resulted in the structure of promoters spanning the GB 2.0 positions 01–12 and terminators, 17–21. But, given the modularity of the approach, any promoter can be combined individually with any 5′-UTR, if parts are designed accordingly (figure 1), with no extra cloning steps. Additionally, we designed superparts: promoter elements including the 5′-segments of coding regions, enabling assembly with coding sequences (CDSs) lacking a start codon and designed to act as fusion partners (spanning GB 2.0 positions 01–13, figure 1A) and providing enhanced stability due to N-terminal amino acid composition determination [11], [13]. In general, the GB design allows fusion of any coding sequence of interest to any N-terminal leader as well as to an array of C-terminal extensions. This might further reinforce the stability of the gene product, as previously demonstrated for the HIV fusion inhibitor, cyanovirin-N [35].

After domestication of a basic set of genetic elements for plastid transformation, we proceeded with the design and assembly of an expression construct. As depicted in figure 1A, a common expression cassette is of a generic structure, basically comprised of the two flanking regions (left targeting region and right targeting region, LTR and RTR, respectively), a transcriptional unit (TU) harboring the selection marker, and another TU encompassing the gene of interest. Virtually any transcriptional unit of choice can be assembled from a set of different elements, either parts or superparts (being fusions of parts). Depending on the size of the part collection, a large number of combinations can be easily designed and cloned in one-pot reactions. In course of this study, we started with a basic set of elements that was steadily growing and further included modules characteristic of diverse species.

To test the functionality of GB assembled chloroplast transformation vectors, we used the sequence encoding griffithsin, a viral entry inhibitor and potential topical prophylactic against HIV infection [36]. Griffithsin has been successfully produced in plants via transient expression systems [37]. Our aim was to further evaluate if tobacco chloroplast expression of this algal gene was feasible.

**Figure 2:**
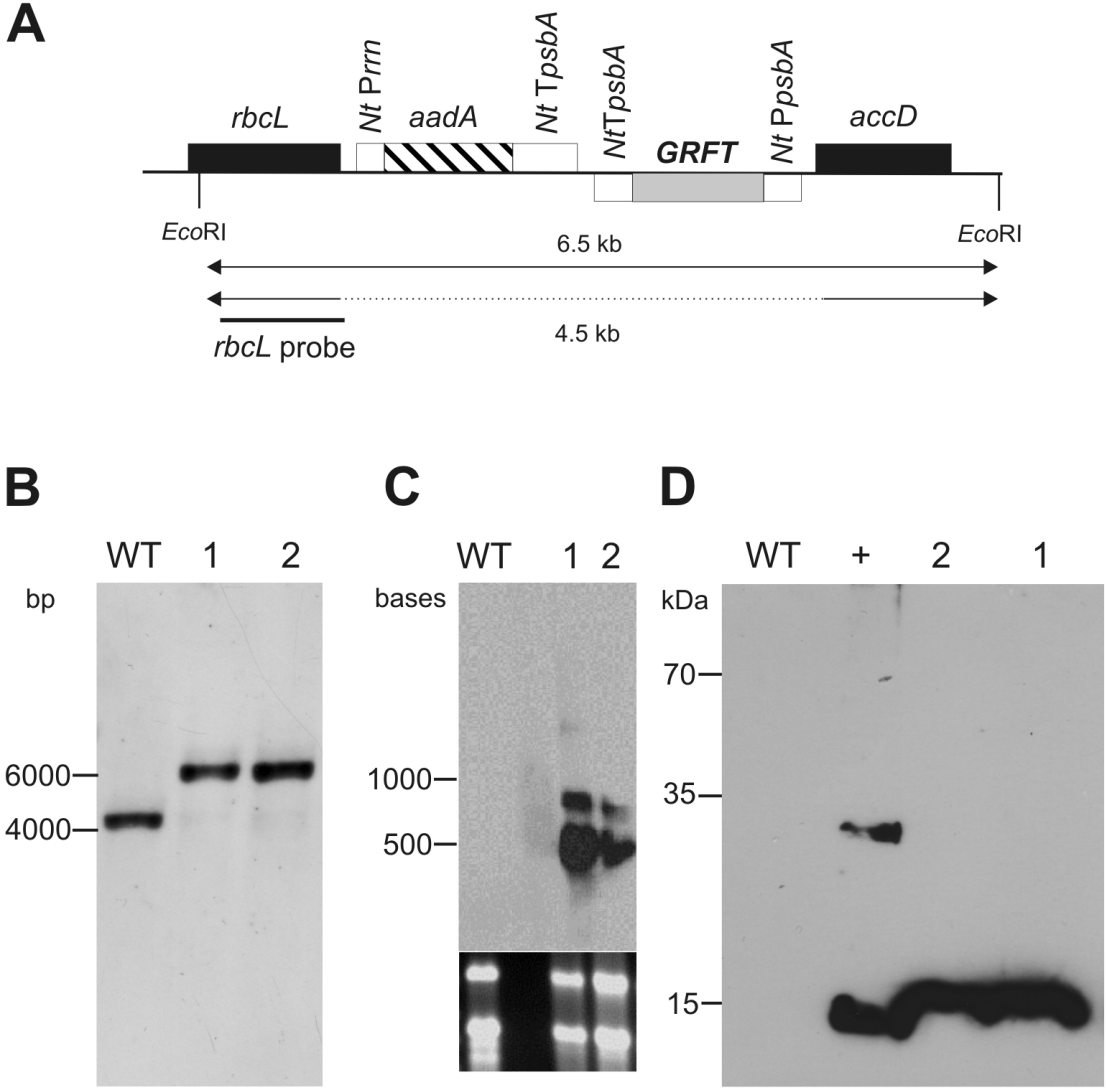
**A)** Schematic representation of the build-up of the expression construct used in this study (not drawn to scale). **B)** Restriction fragment length polymorphism analysis (RFLP) of the DNA isolated from wild type (WT) plants and two transplastomic lines (1 and 2). **C)** Northern blot analysis of the aforementioned lines. The lower panel shows total RNA, while the upper represents transcripts labeled with the *griffithsin*-specific probe. **D)** Western blot analysis of crude protein extracts of WT plants and two transplastomic lines (1 and 2). The blot was probed with an antibody directed against the hexahistidine tag. + represents an extract of a plant containing thioredoxin with a hexahistidine tag, serving as a positive control.

The first TU generated in course of our study was the resistance marker, built from the *aadA* coding sequence together with the *rrn* promoter and the *psbA* terminator from *N. tabacum*. As illustrated in figure 1B, all enumerated modules were taken from the library of standardized parts (in pUPD) and assembled in a GB reaction into pDGB1_α2. In parallel, the LTR was cloned from the pUPD library into pDGB1_α1. The two TUs from the α-level vectors were then combined into pDGB3_Ω1. Similarly, a TU encompassing the griffithsin ORF (open reading frame), P*psbA*, and T*psbA* was assembled in pDGB1_α1R. Using the α1R vector enabled the subsequent combination of relevant TUs in inverse directions, thus preventing the location of two copies of T*psbA* in the same orientation, which might lead to unwelcome homologous recombination events [38]. The griffithsin TU was then combined with the RTR (in pDGB1_α2) into the pDGB3_Ω2 vector. In the final step, the TUs harbored by the pDGB3 Ω1 and Ω2 vectors were combined in the pDGB1_α2 vector, resulting in the ready-to-use transformation vector, shown in figure 2A. Taken together, as depicted in figure 1B, the complete vector was assembled from appropriate parts in seven GB reactions. Since several steps were performed in parallel, only three subsequent reactions were necessary to produce the new vector. As all parts are standardized and reusable, including the TUs and combinations thereof, the presented approach enables generation of a large number of various vectors in only a few additional steps, strongly establishing the power of this modular assembly technology. The fact that the only previously reported endeavors aimed at simplification of plastid transformation vector construction are based on the Gateway recombination cloning system (Life Technologies, Thermo Fisher Scientific) [16], [39] further reinforces the superiority of our standardized approach, as it does not require the use of costly proprietary reagents and the persistently substantial (albeit somewhat reduced) array of intermediary vectors and cloning steps.

With the assembled α-level vector, tobacco leaves were bombarded and transplastomic lines selected on spectinomycin for at least three consecutive regeneration cycles. Two lines were subjected to further investigation. Restriction fragment length polymorphism (RFLP) analysis proved successful integration of the transgene cassette as well as homoplasmy (figure 2B) for both lines (*Eco*RI, 6572 vs. 4423 bp). Northern blot tests confirmed that specific transcripts were generated (figure 2C). The presence of transcripts of larger size than expected showed that, as described earlier [40], read-through transcripts were also produced with the GB assembled cassettes. Since the griffithsin construct was designed to encompass a C-terminal hexahistidine tag, we used western blot with antibodies directed against the tag to detect the recombinant protein in plant extracts. Both lines showed appropriate signals (∼13 kDa), proving that efficient translation of the griffithsin transcripts took place.

## Conclusions

The postulated comprehensive application of the GoldenBraid modular cloning system now affords straightforward and seamless assembly of transcriptional units, coordinated fusions, and multigene operons compatible with the genetic machinery of chloroplasts. Further, it allows reutilization and effortless shuffling of relevant flanking/targeting regions characteristic of not only different parts of a specific plastid genome, but also of diverse representatives of the plant kingdom, thus facilitating easy swapping of species specificity. Moreover, the demonstrated bolstering of the GB toolbox will foster free exchange of the standardized parts between the nuclear- and plastid-specific transformation vectors. The across-the-board compatibility of the GoldenBraid system thus ensured boasts the potential for prospective establishment of an ever-expanding repository of reusable genetic components and bringing together multiple users within the plant synthetic biology scientific community.
